# The Practical Anatomy and Elementary Physiology of the Nervous System

**Published:** 1837-01

**Authors:** 


Art. VI.—*The Practical Anatomy and Elementary Physiology of the Nervous System; designed for the Use of Students in the Dissecting Room.* By F. Le Gros Clark, Demonstrator of Anatomy in St. Thomas's Hospital.—*London,* 1836. 8vo. pp. 367.

We highly approve of the plan which is beginning to be adopted by writers of systematic works on practical anatomy,—we mean that of combining physiological information with descriptive details. This plan not only induces a feeling of pleasure in the mind, and tends to cherish a love and interest for what otherwise would frequently be looked upon with indifference; but it also enables the student to acquire a much more correct idea of the structures described. The work before us is one of the kind we are now alluding to, and, regarded as a whole, is certainly one of the best of its class.

But, in a work of this description, much care ought to be exercised in selecting the kind of physiological opinions introduced into it; particularly when, as in the present instance, it is designed expressly for students. This caution ought more particularly to be attended to where the statements involve theoretical views; and no opinions ought to be given which are not generally received, or which, if new, are not supported by physiological experiments or grounded on positive anatomy. It ought to be remembered that such works, when put into the hands of those who are just commencing the practical study of anatomy, become their textbooks, and almost their only guides in the formation of opinions, and that opinions so acquired, even if they are subsequently proved to be erroneous, are not easily got rid of. Upon this ground we protest, in the strongest manner, against the introduction into the present work of certain physiological views which are anything but well established, and which we have examined somewhat in detail in another part of our present number; we allude to the views respecting the *excito-motory* system of Dr. Marshall Hall. Mr. Clark is almost the only anatomical writer who appears to have received these ingenious views without some distrust of their correctness; but, at any rate, we are surprised that he should have incorporated them, unconfirmed as they are by any other physiological authority, into a book of descriptive anatomy intended expressly for students. Instead of rendering the knowledge of the nervous system more intelligible to the student, it will rather tend to confound and perplex him.

That part of Mr. Clark's work which is more particularly his own,—we mean the descriptive anatomy of the nervous system,—deserves an unqualified commendation, and cannot fail to be of great use to the student in his dissections of this most difficult part of anatomy. A work devoted exclusively to the descriptive anatomy of the nerves was much wanted for the dissecting-room, and we think that Mr. Clark has executed his task with great ability. The literary portion of the work is highly creditable to his scholarship; and we venture confidently to anticipate, from this specimen of his powers, a career honorable to himself and advantageous to his profession.

